# Expenditures on Screening Promotion Activities in CDC’s Colorectal Cancer Control Program, 2009–2014

**DOI:** 10.5888/pcd16.180337

**Published:** 2019-06-06

**Authors:** Florence K. L. Tangka, Sujha Subramanian, Sonja Hoover, Maggie Cole-Beebe, Amy DeGroff, Djenaba Joseph, Sajal Chattopadhyay

**Affiliations:** 1 Centers for Disease Control and Prevention, Atlanta, Georgia; 2 RTI International, Waltham, Massachusetts

## Abstract

**Introduction:**

The Centers for Disease Control and Prevention (CDC) established the Colorectal Cancer Control Program (CRCCP) in 2009 to reduce disparities in colorectal cancer screening and increase screening and follow-up as recommended. We estimate the cost for evidence-based intervention and non–evidence-based intervention screening promotion activities and examine expenditures on screening promotion activities. We also identify factors associated with the costs of these activities.

**Methods:**

By using cost and resource use data collected from 25 state grantees over multiple years (July 2009 to June 2014), we analyzed the total cost for each screening promotion activity. Multivariate analysis was used to assess the factors associated with screening promotion costs reported by grantees.

**Results:**

The promotion activities with the largest allocation of funding across the years and grantees were mass media, patient navigation, outreach and education, and small media. Across all years of the program and across grantees, the amount spent on specific promotion activities varied widely. The factor significantly associated with promotion costs was region in which the grantee was located.

**Conclusion:**

CDC’s CRCCP grantees spent the largest amount of the screening promotion funds on mass media, which is not recommended by the Community Preventive Services Task Force. Given the large variation across grantees in the use of and expenditures on screening promotion interventions, a systematic assessment of the yield from investment in specific promotion activities could better guide optimal resource allocation.

**Summary**

**What is already known on this topic?**

Colorectal Cancer Control Program grantees spent most of their funding on interventions recommended by the Community Guide. However, a third of grantees’ funding was spent on interventions not recommended by the Community Guide.

**What is added by this report?**

Our results update previous estimates and provide data on the resources expended and the factors associated with using evidence-based interventions recommended by the Community Guide.

**What are the implications for public health practice?**

These findings will support future colorectal cancer program planning to ensure that resources are used to implement evidence-based interventions. Economic evaluations inform future scale-up and improve the efficiency of colorectal cancer screening programs to achieve the Healthy People 2020 objective.

## Introduction

Colorectal cancer (CRC) screening can reduce the burden of this disease and is recommended in guidelines (1–3). Analysis of data from the Behavioral Risk Factor Surveillance System showed that the prevalence of having had CRC screening was 67.3% (4), lower than the Healthy People 2020 goal of 70.5% and the National Colorectal Cancer Roundtable goal of 80% by 2018 (5). Even though the use of CRC screening tests has increased, screening use is lower among certain populations, such as the uninsured and those with less than a high school education (6). To reduce these disparities and increase quality screening and appropriate follow-up for CRC, the Centers for Disease Control and Prevention (CDC) established the Colorectal Cancer Control Program (CRCCP) in 2009. CDC provided funding and technical assistance to 29 grantees (25 states and 4 tribal organizations) to increase CRC screening through population-level, evidence-based interventions (EBIs) and provide direct CRC clinical screening services to low-income uninsured and underinsured adults aged 50 to 64 years (7). EBIs are activities recommended by the Community Preventive Services Task Force (Community Guide) to increase CRC screening test use and include client reminders, provider reminders, provider assessment and feedback, and reducing structural barriers (8). Non-EBIs are screening promotion activities that were selected and used by grantees but have not been recommended by the Community Guide.

The objective of this study was to update the previous report (9) by using 5 years (2009–2014) of data to estimate the cost for EBI and non-EBI screening promotion activities and to examine expenditures on screening promotion activities. Results from this study will provide the economic basis to understand the resources expended on EBIs recommended by the Community Guide and evaluate the factors associated with the use of EBIs. These findings will support future CRC program planning to ensure that resources are used for implementing EBIs. Economic evaluations are essential to inform future scale-up and improve the efficiency of CRC screening programs to achieve the Healthy People 2020 objective.

## Methods

Data were collected about grantees’ expenditures for activities by using a web-based cost assessment tool (CRCCP-CAT). The design of the CRCCP-CAT was based on previously published methods to collect activity-based cost data for program evaluation (10–13). Grantee staff were trained to use the web-based CRCCP-CAT via webinars, a user’s guide, and technical assistance. The web-based version of the tool allowed for real-time data collection. Because of embedded data checks, the quality of data reporting was also higher than prior testing with a Microsoft Excel–based instrument (14). Staff from CRCCP-funded grantees completed the CRCCP-CAT annually from July 2009 through June 2014.

By using the CRCCP-CAT, grantees provided information on funding from all sources: CDC, other federal, nonfederal, and in-kind contributions. Grantees reported on the following budget categories: staff salaries, contract expenditures, purchases of materials and equipment, and administration or overhead costs. Costs and resources used were then allocated to specific grantee activities related to screening promotion, screening provision, and overarching activities that supported both screening promotion and provision activities; all labor and nonlabor costs were assigned to the specific activities performed by the grantees (Box).

**Box. Screening Promotion, Screening Provision, and Overarching Components of the Colorectal Cancer Control Program, 2009–2014**

**Screening Promotion Activities**

- Client reminders
- Small media
- Provider assessment and feedback
- Provider reminders
- Reducing structural barriers (including patient navigation)
- Mass media
- Reducing out-of-pocket cost
- Enrolling in insurance programs
- Outreach, education, and incentives
- Patient navigation and support
- Other promotion activities

Five screening promotion activities (client reminders, small media, provider assessment and feedback, provider reminders, and reducing structural barriers) are evidence-based interventions and supporting activities recommended by the Community Preventive Services Task Force and published in the *Guide to Community Preventive Service*s for increasing colorectal cancer screening compliance using fecal occult blood tests.

**Screening Provision Activities**

- Provider contracts, billing systems, other billing procedures
- Patient navigation and support
- Screening and diagnostic services (only labor, if any are reported)
- Ensure cancer treatment
- Other screening provision activities
- Screening and diagnostic services (only clinical)
- Screening and diagnosis
- Surveillance

**Overarching Components Activities**

Overarching components relate to both screening promotion and screening provision activities.

- Program management
- Quality assurance and professional development
- Partnership development and maintenance
- Clinical and cost data collection and tracking
- Program monitoring and evaluation
- Administration
- Other activities

Costs were aggregated and analyzed for screening promotion activities, both EBIs and non-EBIs, across 25 state grantees for multiple years. Screening promotion activities included client reminders, small media (15,16), mass media, outreach and education, provider assessment and feedback, patient navigation, and other promotion activities. The “other” promotion activities category accounted for only a small proportion of the expenditures and were pooled together for analysis. The activities were EBIs recommended by the Community Guide, such as provider reminders and reducing structural barriers (eg, modifying health center times, offering services in nonclinical settings). Additional activities included reducing out-of-pocket costs, enrolling patients in Medicaid or other private or public insurance, and other miscellaneous activities.

We created a panel data set that included 1 record for each grantee for each year of data submission. We analyzed the total cost for each screening promotion activity by grantees’ state-level screening prevalence: high (screening rates ranging from 69.6 to 76.6 [>66th percentile]), medium (screening rates ranging from 65.9 to 69.5 [34th to 66th percentile]), and low (screening rates ranging from 56.5 to 65.8 [<34th percentile]). We also analyzed the total cost by grantees’ populations eligible for screening based on percentiles (<34th percentile, 34th to 66th percentile, or >66th percentile), using an appropriate age range (age 50–75) for those eligible but not screened. We hypothesized that the baseline level of screening compliance and the total number of individuals eligible for screening might affect the resources expended on specific interventions. For example, while often a high-cost intervention, grantees may consider mass media when there is a large volume of unscreened individuals and low levels of screening compliance, given mass media’s potential large reach. The Community Guide statement on mass media acknowledges that it will likely not have a meaningful impact when screening prevalence is high because of ceiling effects and the expectation that mass media would have a limited ability to address unresolved barriers among people who remain unscreened (17). We used the 2012 Behavioral Risk Factor Surveillance System (BRFSS) CRC screening measures (based on multiple tests recommended) to assess screening prevalence during the midpoint of the implementation of the CRCCP (July 2009 to June 2014) and population counts for grantee states from the 2012 American Community Survey to calculate the number of people eligible for CRC screening (people aged 50 to 75 years). Lastly, we looked at various characteristics (eg, geographic location, size of population eligible for screening, screening prevalence), comparing grantees that used mass media and those that did not. On the basis of a previous analysis (9), mass media was one of the most expensive interventions undertaken by the CRCCP. This analysis was undertaken to understand, among other things, whether there were specific characteristics associated with mass media use. All costs include in-kind contributions and were adjusted by using the employment cost index (18).

The total number of records available for analysis comprised 121 grantee years. We excluded the 4 tribal organization grantees from all years of our analysis because screening data for tribes or tribal organizations are not available through BRFSS, and we did not have accurate estimates of their eligible population (age 50–75 years). In addition, 3 grantees were not included in Year 1 because they did not implement the program until Year 2, and 1 grantee was excluded from Year 2 because it did not report screening promotion costs.

We provide descriptive analyses on grantee characteristics, mass media use, and award amounts. We used multivariate analysis to assess the factors associated with screening promotion costs reported by grantees; we ran a random effects model to account for the panel database that consisted of multiple years of data for each grantee. We examined both the cost of screening promotion and the percentage of total cost allocated to client-related and provider-related EBIs recommended by the Community Guide (Box). We used the log transformation of the dependent cost variable to account for the skewness in the distribution of promotion cost across the grantees. We estimated a random effects equation with grantee characteristics as explanatory variables. Grantee characteristics include region, population size, and screening prevalence. To avoid bias when interpreting the estimated coefficients, we used Duan smearing retransformation on the log-transformed dependent variable, promotion cost, and estimated 95% confidence intervals by using a bootstrapping technique (19). The same overall model specification was used to examine total promotion cost and the proportion of total funding allocated by each grantee to EBIs.

## Results

**Figure 1:**
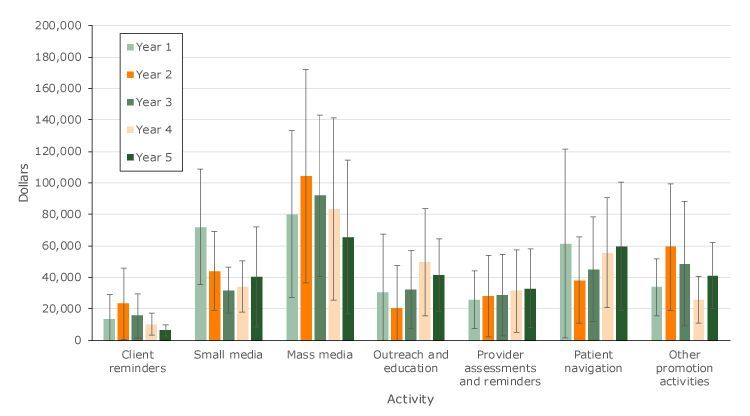
Average cost per grantee for each screening promotion activity, by year, Colorectal Cancer Control Program, 2009–2014. Error bars represent 95% confidence intervals.

Mass media was the largest cost category for all years of the CRCCP, with costs ranging from $65,453 to $104,351(Figure 1), and it comprised approximately 28% of funds spent on screening promotion. The client reminders category was most often the lowest cost category across years, with costs ranging from $6,241 to $23,350. Overall, across grantees and across all years of the program, the amount spent on specific promotion activities varied greatly, as evidenced by the large 95% confidence intervals. For example, in Year 1, the grantees spent a substantially larger proportion on small media than in any other subsequent program year. The highest costs for screening promotion intervention were mass media, patient navigation, outreach and education, and small media.

**Table:** 

Category	Year 1 (95% CI)	Year 2 (95% CI)	Year 3 (95% CI)	Year 4 (95% CI)	Year 5 (95% CI)
Client reminders	13,357 (0–28,963)	23,350 (638–46,062)	15,520 (1,633–29,407)	10,175 (3,094–17,257)	6,241 (2,814–9,668)
Small media	71,769 (35,098–108,440)	43,978 (18,848–69,107)	31,661 (16,998–46,324)	34,012 (17,828–50,197)	40,191 (8,683–71,700)
Mass media	79,962 (27,077–132,847)	104,351 (36,539–172,163)	91,825 (40,600–143,049)	83,375 (25,632–141,119)	65,453 (16,388–114,517)
Outreach and education	30,062 (0–67,058)	20,521 (0–47,606)	31,941 (7,181–56,701)	49,662 (15,795–83,530)	41,300 (18,195–64,406)
Provider assessments and reminders	25,502 (7,201–43,802)	28,301 (2,401–54,200)	28,710 (2,889–54,532)	31,355 (5,263–57,447)	32,909 (7,857–57,962)
Patient navigation	61,364 (1,491–121,237)	38,185 (10,720–65,650)	45,123 (11,936–78,310)	55,451 (20,550–90,351)	59,731 (19,114–100,348)
Other promotion activities	33,812 (15,786–51,838)	59,243 (18,970–99,516)	48,669 (9,045–88,294)	25,720 (10,740–40,699)	40,964 (19,950–61,978)

Overall, 19% of grantees were in the South, 25% in the Northeast, 20% in the Midwest, and 36% in the West (Table 1). A higher proportion of grantees in the South used mass media than grantees in other regions, while the Northeast had the lowest proportion of grantees to use mass media. The average award ranged from $870,747 in the West to $1,614,766 in the Northeast.

**Table 1:** Grantee Characteristics, Mass Media Use, and Average Award, Colorectal Cancer Control Program, 2009–2014

Category	Overall, %	Grantees That Used Mass Media, %	Grantees That Did Not Use Mass Media, %	Average Award (including In-kind), Mean $ (95% CI)
**Region**				
South	19	27	6^a^	1,062,337 (903,115–1,221,559)
Northeast	25	18	36^b^	1,614,766 (1,095,380–2,134,152)
Midwest	20	24	13	970,045 (864,198–1,075,891)
West	36	31	45	870,747 (796,273–945,221)
**Size of population eligible for screening**				
Small (228,339–736,635)	33	28	40	967,876 (863,195–1,072,558)
Medium (854,624–1,618,255)	32	36	26	938,551 (849,482–1,027,621)
Large (1,749,719–9,472,316)	35	35	34	1,447,336 (1,111,326–1,783,346)
**Screening Prevalence**				
Low (56.5–65.8)	36	32	43	855,663 (788,107–923,219)
Medium (65.9–69.5)	31	35	26	1,421,591 (999,269–1,843,913)
High (69.6–76.6)	32	32	32	1,097,463 (991,600–1,203,326)

Abbreviation: CI, confidence interval.

a *P* < .001.

b *P* < .05.

The size of the population eligible for screening was similar for grantees overall and between those using mass media and those not (Table 1). Approximately 33% of grantees served areas with a small population eligible for screening, 32% with a medium eligible population, and 35% with a large eligible population. Of grantees using mass media, 36% were in areas with a medium eligible population; of grantees not using mass media, 40% were in areas with small eligible populations. The average award ranged from $938,551 for grantees serving areas with a medium population to $1,447,336 for grantees serving areas with a large population.

Estimates of screening prevalence did not vary much between those using and those not using mass media (Table 1). Overall, 36% of grantees serve areas with low screening prevalence, 31% serve areas with medium screening prevalence, and 32% serve areas with high screening prevalence. Of grantees using mass media, 35% are in areas of medium screening prevalence; of grantees not using mass media, 43% are in areas of low screening prevalence. The average award ranged from $855,663 in areas with a low screening prevalence to $1,421,591 in areas with a medium screening prevalence.

**Figure 2:**
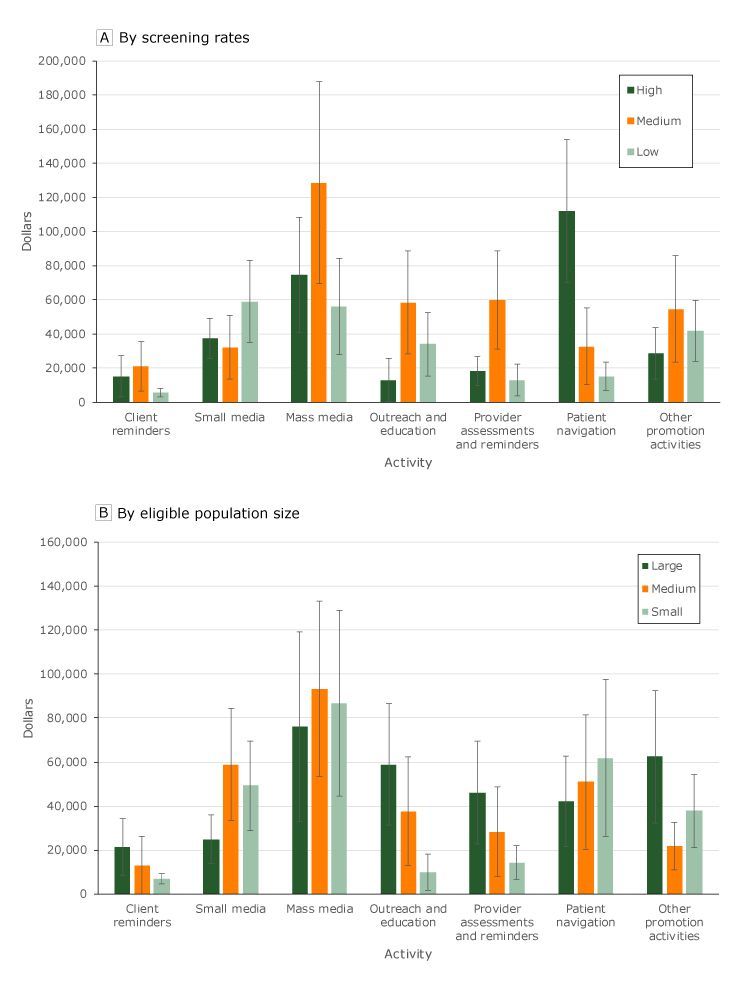
Average cost, in dollars, for each screening promotion activity (5-year period), by screening rates and by eligible population size, Colorectal Cancer Control Program, 2009–2014. Error bars represent 95% confidence intervals. State-level screening rates (panel A) were classified as high (screening rates ranging from 69.6 to 76.6 [>66th percentile]), medium (screening rates ranging from 65.9 to 69.5 [34th to 66th percentile]), or low (screening rates ranging from 56.5 to 65.8 [<34th percentile]). Grantee populations eligible for screening (panel B) were analyzed based on percentiles (small, <34th; medium, 34th–66th; large, >66th percentile) for those eligible but not screened.

Grantees with high screening prevalence spent the most on patient navigation ($111,764) compared with grantees with medium ($32,746) and low ($15,248) screening prevalence (Figure 2, panel A). Alternatively, grantees with medium screening prevalence spent the most on mass media ($128,527), while grantees with low screening prevalence spent the most on small media ($59,066). Grantees with high screening prevalence spent the least on outreach and education, while grantees with low screening prevalence spent the least on client reminders. Grantees with large, medium, and small populations eligible for screening spent most on mass media ($76,240, $93,311, and $86,635, respectively) (Figure 2, panel B). All grantees, regardless of the size of their population eligible for screening, spent the least on client reminders.

**Table:** 

Category	High (95% CI)	Medium (95% CI)	Low (95% CI)
**By screening rates**			
Client reminders	15,282 (3,236–27,328)	21,006 (6,502–35,509)	5,874 (3,283–8,465)
Small media	37,430 (25,826–49,033)	32,167 (13,519–50,814)	59,066 (34,931–83,202)
Mass media	74,774 (40,957–108,591)	128,527 (69,446–187,608)	56,357 (28,175–84,538)
Outreach and education	13,010 (379–25,641)	58,501 (28,241–88,760)	34,001 (15,170–52,832)
Provider assessments and reminders	18,362 (9,859–26,864)	59,854 (31,088–88,620)	13,047 (3,774–22,320)
Patient navigation	111,764 (69,905–153,623)	32,746 (10,348–55,144)	15,248 (7,143–23,352)
Other promotion activities	28,798 (13,702–43,893)	54,679 (23,508–85,851)	42,013 (24,226–59,801)

**Table:** 

Category	Large (95% CI)	Medium (95% CI)	Small (95% CI)
**By eligible population size**			
Client reminders	21,420 (8,315–34,526)	12,735 (0–26,310)	6,984 (4,750–9,218)
Small media	24,876 (13,895–35,857)	58,954 (33,449–84,459)	49,383 (29,017–69,748)
Mass media	76,240 (32,978–119,503)	93,311 (53,259–133,362)	86,635 (44,334–128,936)
Outreach and education	58,944 (31,490–86,398)	37,580 (12,929–62,232)	9,898 (1,768–18,029)
Provider assessments and reminders	46,206 (22,771–69,642)	28,343 (7,886–48,799)	14,363 (6,747–21,978)
Patient navigation	42,163 (21,532–62,793)	50,949 (20,487–81,411)	61,818 (26,103–97,533)
Other promotion activities	62,432 (32,371–92,494)	21,822 (11,060–32,584)	37,809 (21,235–54,383)

In the regression estimating the regionally adjusted promotion cost, region was still a significant determinant of promotion costs even after we adjusted for regional differences in employment costs (Table 2). We found that grantees in the West have, on average across all years, a $236,051 lower promotion cost than those in the South.

**Table 2:** Regression Estimates for Cost of Screening Promotion and Proportion of Total Cost Allocated to Evidence-based Interventions, Colorectal Cancer Control Program, 2009–2014

Category	Total Cost of Screening Promotion, Estimate $ (95% CI)	Proportion of Total Cost Allocated to Client-related and Provider-related Evidence-based Strategies Recommended by the Community Guide, Estimate (95% CI)^a^
**Region **		
South	1 [Reference]	
Northeast	−25,965 (−229,222 to 324,123)	0.145 (−0.082 to 0.372)
Midwest	−110,866 (−276,887 to −179,093)	−0.051 (−0.275 to 0.174)
West	−236,051^b^ (−345,123 to −55,084)	0.245^b^ (0.033 to 0.457)
**Size of population eligible for screening **		
Small (228,339–736,635)	1 [Reference]	
Medium (854,624–1,618,255)	−26,755 (−195,041 to 231,280)	0.036 (−0.141 to 0.214)
Large (1,749,719–9,472,316)	20,025 (−171,755 to 320,351)	0.124 (−0.063 to 0.310)
**Screening Prevalence**		
Low (56.5–65.8)	1 [Reference]	
Medium (65.9–69.5)	154,797 (−85,528 to 530,999)	−0.129 (−0.315 to 0.058)
High (69.6–76.6)	−107,439 (−265,727 to 153,173)	0.075 (−0.133 to 0.282)

Abbreviation: CI, confidence interval.

a Coefficients were estimated using Duan smearing retransformation.

b *P* < .05.

The second regression estimated the percentage of regionally adjusted costs allocated toward client-related and provider-related EBIs recommended by the Community Guide. These activities included client reminders, small media, one-on-one education, reducing structural barriers, and provider assessment and feedback. Grantees in the West allocated a significantly greater percentage of total costs to Community Guide–recommended activities relative to grantees in the South (25%) (Table 2).

## Discussion

Findings from the analysis of 5 years of cost data show that CRCCP grantees spent much of the screening promotion funds on interventions recommended by the Community Guide. We saw large variations across grantees in the amount spent on each promotion activity. The top screening promotion activities, excluding the “other” category, to which resources were allocated were mass media, patient navigation, outreach and education, and small media. Across the 5 years, grantees allocated more resources to small media in the first year of the program. Small media might be easier to implement while other interventions might require more planning; CDC and other organizations provide small media materials that grantees can easily tailor to implement targeted campaigns.

The highest cost category across the 5 years was mass media, an intervention for which the Community Guide did not have sufficient evidence to make a recommendation. Beyond general use of mass media campaigns (18 of 29 grantees in year 5), the share of screening promotion funds expended for this activity is high. Advertisements on television, radio, and billboards are more expensive than print materials such as letters and brochures for small media. This finding is consistent with findings reported in prior studies (9). Given the resources required for this screening promotion activity and its widespread use, it is critical to evaluate the effectiveness and cost-effectiveness of mass media to provide the evidence base to guide future decisions about resource allocation.

We also found differences in the allocation of funds to promotion interventions by levels of screening prevalence and the size of the population eligible for screening. Grantees in areas with a screening prevalence of 69.6% or higher allocated the smallest proportion of their screening promotion funds to outreach and education compared with grantees in areas with a screening prevalence of 69.5% or lower. The priority for grantees in areas with higher screening prevalence is navigating the patients along the screening continuum to ensure adherence with recommended screening, diagnostic follow-up, and referral for treatment recommendations; thus, these grantees spent more resources on patient navigation. All grantees expended fewer resources on client reminders than any other promotion activity. It is possible that clinics integrated client reminders into their electronic health record systems and bear the burden of any related costs. Regional variation existed in total allocation of resources to screening promotion activities and in the proportion of resources allocated to EBIs. Grantees in the South showed significant differences from those in the West. We hypothesize that there could be numerous potential reasons for this variation, including proportion of minorities served, which could result in cultural differences that may have affected selection of screening promotion interventions to target specific groups. Further assessments should explore the reasons for the regional variation.

Our study has several limitations. The grantees reported cost data retrospectively, which might result in misallocation of resources and errors in cost estimates. To reduce such errors, all grantees were provided a standardized data collection tool and user guides with activity definitions, training, and ongoing technical assistance. The cost assessment tool used in this study was previously tested and validated; several of the programs were already familiar with the tool. Our regression model was constrained by the small sample size (121 observations), which limits the number of explanatory variables we could include in the model. As a result, other unmeasured factors, in addition to the grantees’ geographic region and the screening prevalence, could significantly influence screening promotion cost. We also found large variation in the cost across grantees, and a larger sample size would have allowed us to more fully explore these differences.

Nevertheless, our study provides a few lessons and reveals some additional gaps in the implementation economics literature. Small media was often used at the initiation of the program, and this could be due to the availability of small media materials (20) and tools that grantees could easily tailor. Standardized guidelines and tool kits for other types of EBIs should be made available so that they can be quickly and easily implemented, potentially saving time and money. Although the Community Guide did not yet have the evidence needed to recommend the use of mass media for CRC screening promotion, many grantees used this intervention, which suggests mass media has perceived value. Mass media, primarily television, has been effective in preventing tobacco use, a risk factor for tobacco-related chronic diseases (21). Results from analysis of benefits and costs of CDC’s Screen for Life: National Colorectal Cancer Action Campaign (SFL) suggest that the SFL campaign might have contributed to improving CRC screening rates at a minimal cost (16). Additional studies are needed to evaluate mass media’s impact and cost-effectiveness and the decision-making process of grantees in selecting to use mass media.

Given the large variation across grantees on screening promotion interventions, a systematic assessment of needs matched with promotion activities, and their impact on screening rates, could provide better guidance on optimal resource allocation. In addition to the EBIs recommended by the Community Guide, grantees are using other interventions (eg, patient and provider incentives). CDC is applying these lessons learned to its study of the currently funded CRCCP grantees (15). Our ongoing study examines the cost-effectiveness of these interventions, those that are recommended by the Community Guide as well as those for which there is insufficient evidence to make a recommendation. Findings from the ongoing implementation economics studies will contribute to the evidence base for the optimal mix of cost-effective screening promotion activities and strategies that grantees can use to increase CRC screening rates. These strategies might also inform efforts to address other cancer screening programs.
